# Supplementary material for: Evolutionary Flexibility of Ribosome Biogenesis in Bacteria
Source: Mol Biol Evol. 2025 Nov 24;42(11):msaf288. doi: 10.1093/molbev/msaf288 (PMC12641178; doi:10.1093/molbev/msaf288)
Supplement: msaf288_Supplementary_Data [file msaf288_supplementary_data.zip › MBErevision2_v251005_Supplementary_information.pdf]

Supplementary Information for:

Evolutionary Flexibility of Ribosome Biogenesis in Bacteria

Kazuaki Amikura[a,1], Shun'ichi Ishii[b], Yoshihiro Shimizu[c], and Shino Suzuki[a,b,d,1]

a Geobiology and Astrobiology Laboratory, RIKEN Pioneering Research Institute (PRI), Wako, Saitama, Japan; b Institute for Extra-cutting-edge Science and Technology Avant-garde Research (X-star), Japan Agency for Marine and Earth Science and Technology (JAMSTEC), Yokosuka, Kanagawa, Japan; c Laboratory for Cell-Free Protein Synthesis, RIKEN Center for Biosystems Dynamics Research (BDR), Kobe, Hyogo, Japan; d Institute of Space and Astronautical Science (ISAS), Japan Aerospace Exploration Agency (JAXA), Sagamihara, Kanagawa, Japan

1To whom correspondence should be addressed.

E-mail: kazuaki.amikura@riken.jp and shino.suzuki@riken.jp

Supplementary Table 1.

This table presents the curated list of RBFs used in this study. KEGG_ID refers to the KEGG orthology ID. Name indicates the commonly used name of the factor, while Synonym lists any alternative names. Category in this study describes the classification or role of each factor based on the current research. Category in KEGG Pathway (Ribosome biogenesis in prokaryotic type) refers to the corresponding category in the KEGG database. Essentiality (PEC) and Essentiality (KEIO) columns reflect the factor's essentiality as defined by the Profiling of E.coli Chromosome (PEC, https://shigen.nig.ac.jp/ecoli/pec/) and the KEIO collection, respectively[8,51]. BAC120 and LBCA indicate the presence of the factors in the BAC120 phylogenetic marker set and in the last bacterial common ancestor (LBCA), respectively[11,52]. References provide key literature for RBFs that isn't included in the KEGG database, and the Note column contains any additional relevant annotations.

Supplementary Table 2.

The table shows the number and percentage of genomes assigned to each class within Patescibacteria in GTDB r226 (representative genomes only) and in the 505 genomes analyzed in this study. Classes not highlighted in blue were grouped as "others" in our study.

Supplementary Table 3.

This table presents ribosome biogenesis factors and their conservation across CPR and Non-CPR bacterial lineages. KEGG_ID refers to the KEGG orthology ID associated with each factor. symbol denotes the gene symbol commonly used for each factor. Category describes the functional classification. non-CPR (%) and CPR (%) indicate the conservation of each factor within Non-CPR and CPR bacteria, respectively.

Supplementary Table 4.

This table displays the mutual information (MI) values for gene pairs (Gene A and Gene B) and the distribution of species based on the presence or absence of these genes in CPR bacteria.

Supplementary Table 5.

This set of tables presents the number of gene interactions (edges) and the total mutual information (MI) for each gene, calculated at three different MI thresholds: greater than 0.1, 0.05, and 0.025. The genes are ranked in descending order based on their total MI values for each threshold.

Supplementary Data 1

This file provides detailed information on CPR genomes. Each column is described as follows. Assembly Accession: The unique accession number assigned to each genome assembly. Organism Name: The taxonomic name of the organism based on genome data. Rep. genome: Indicates whether the genome is a complete representative genome (Comp) or partial. Sum of tRNAs: The total number of tRNA genes identified in each genome. Completeness: The estimated completeness by CheckM2 of each genome, expressed as a percentage. Contamination: The estimated level of contamination by CheckM2 in each genome, expressed as a percentage. Size: Genome size in megabases (Mb). GC: GC content percentage of the genome. Scaffolds: The number of scaffolds in each genome assembly. WGS: The Whole Genome Shotgun (WGS) project identifier. ncbi_organism_name: The organism name as recorded in the NCBI database. ncbi_taxonomy: The taxonomy classification according to NCBI. gtdb_taxonomy: The taxonomy classification according to the Genome Taxonomy Database (GTDB). GTDB_Phylum: The phylum classification based on GTDB taxonomy. GTDB_Class: The class classification based on GTDB taxonomy. Group: Classification group of CPR bacteria in this study.

Supplementary Data 2

This file provides detailed information on non-CPR genomes. Each column is described as follows. Assembly Accession: The unique accession number assigned to each genome assembly. Organism Name: The taxonomic name of the organism based on genome data. Completeness: The estimated completeness by CheckM2 of each genome, expressed as a percentage. Contamination: The estimated level of contamination by CheckM2 in each genome, expressed as a percentage. Size: Genome size in megabases (Mb). GC: GC content percentage of the genome. Scaffolds: The number of scaffolds in each genome assembly. nonCPR107: Indicates whether the genome is part of nonCPR107 as shown in Figure 3A. symbiotic/parasitic: Indicates manually selected symbiotic or parasitic bacteria.

Supplementary Data 3

Alignment of the 23S rRNA seed sequences from Rfam with the 23S rRNA sequences of CPR bacteria, with regions corresponding to helices H76, H77, and H78 extracted and presented in FASTA format.

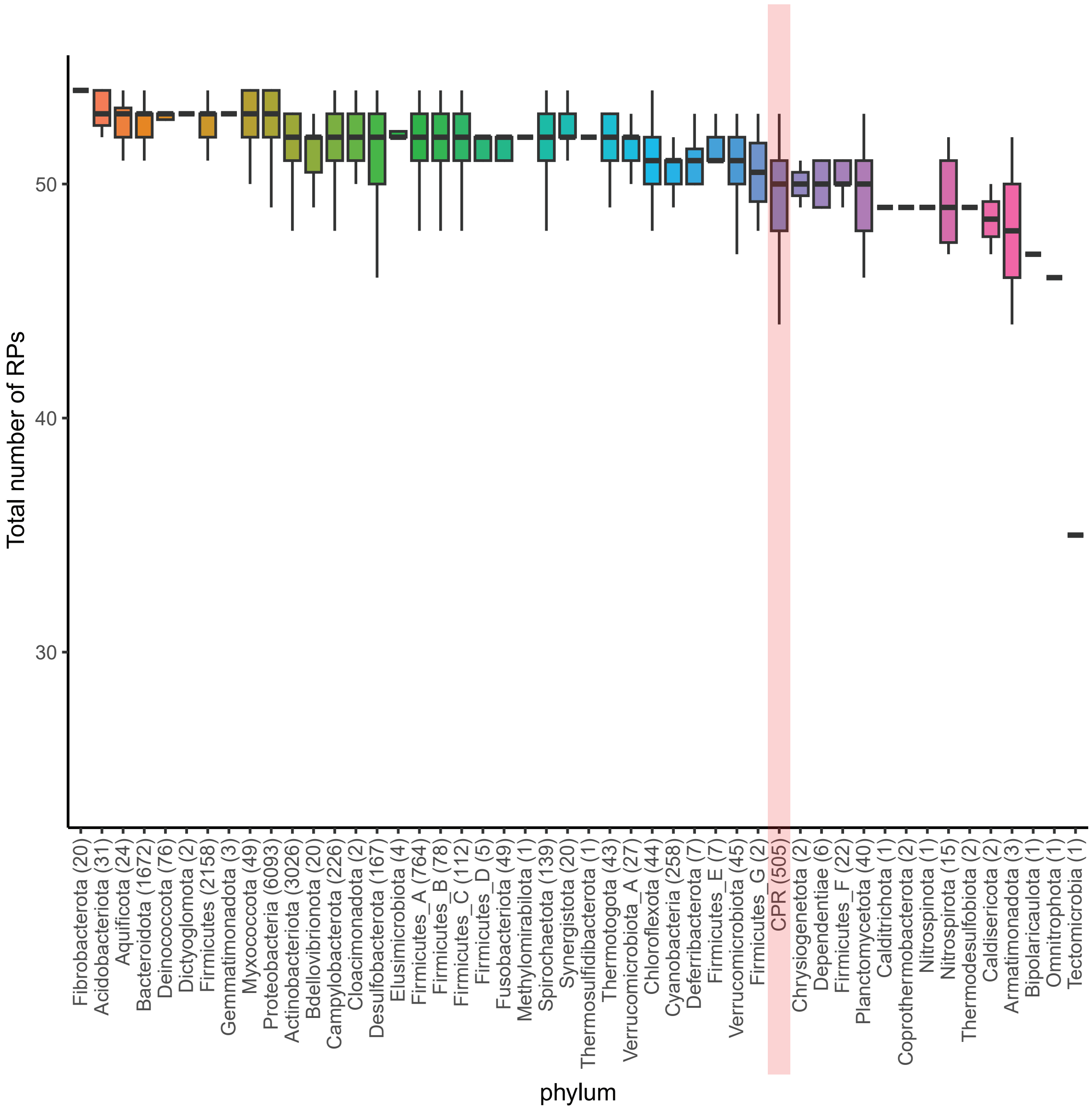

Supplementary Fig 1: Boxplot representation of the number of RPs across different phyla in bacteria. Firmicutes divided into seven groups in the AnnoTree database.

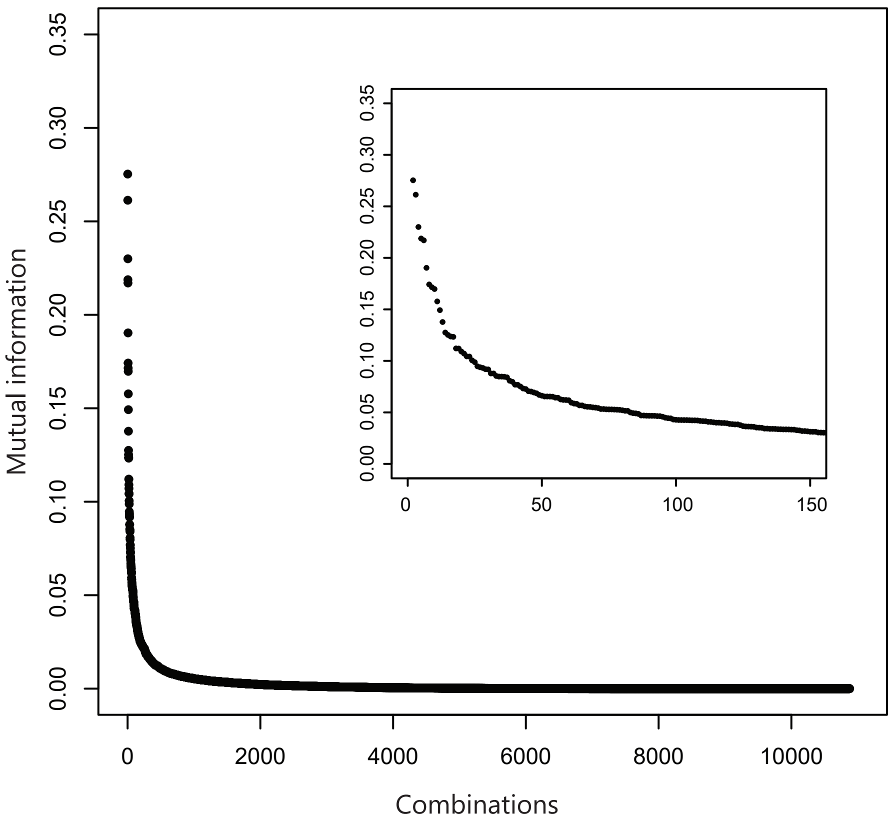

Supplementary Fig 2: The combinations and the mutual information value. Small graph show different x-axis ranges.

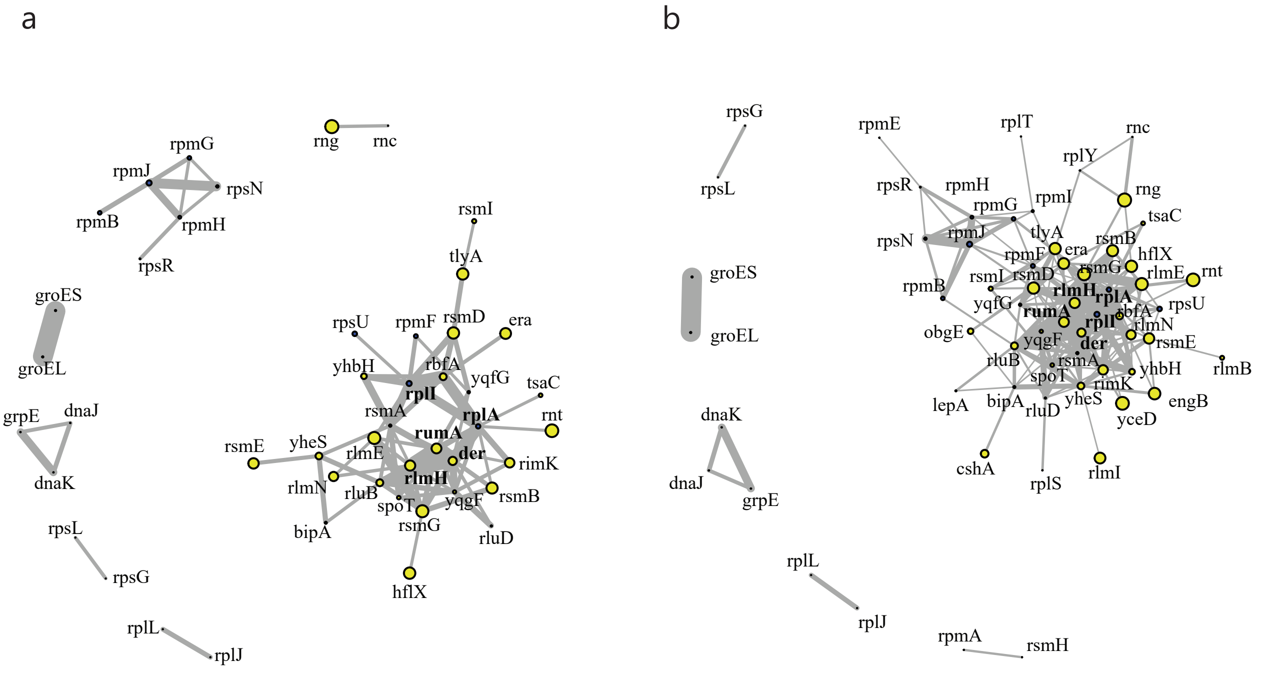

Supplementary Fig 3: a, The dependency network when the mutual information threshold is 0.05. **b,** The dependency network when the mutual information threshold is 0.025.

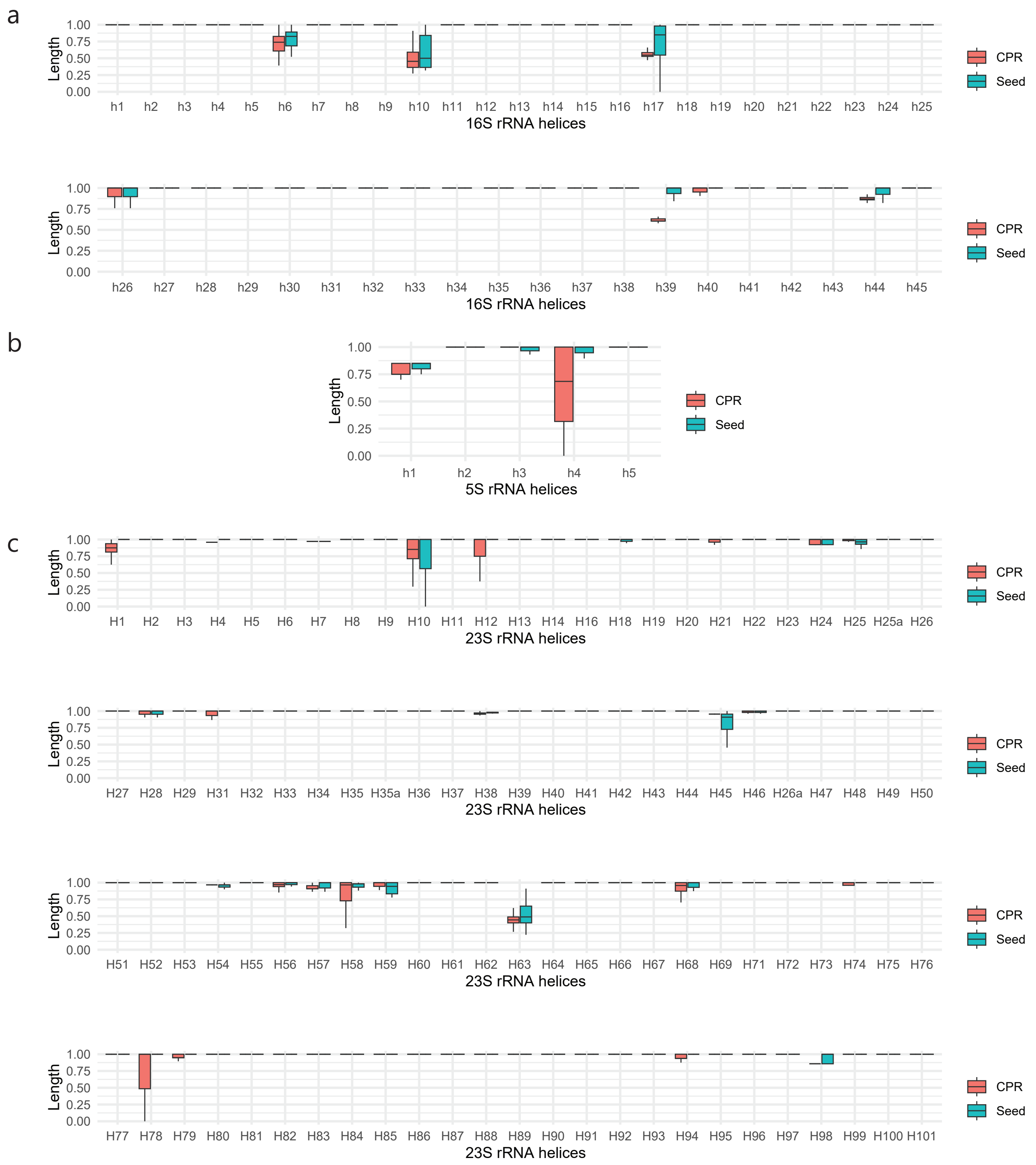

Supplementary Fig 4: a-c,The boxplots represent the median length of helices. (**a**) 16S rRNA (**b**) 5S rRNA (**c**) 23S rRNA.

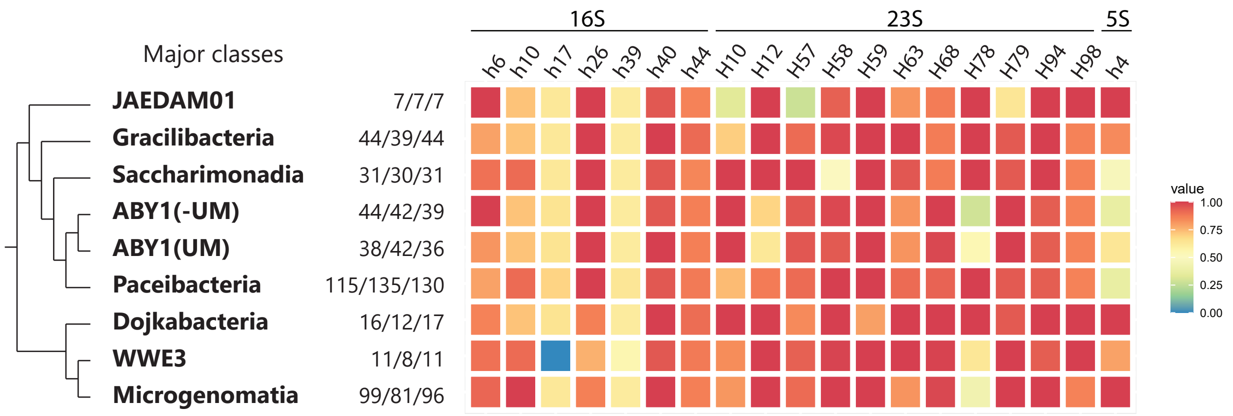

Supplementary Fig 5: Truncated rRNA structures in CPR bacteria. The heatmap represents the median length of helices. The numbers next to the heatmap shows the number of sequences from 16S/23S/5S rRNA.

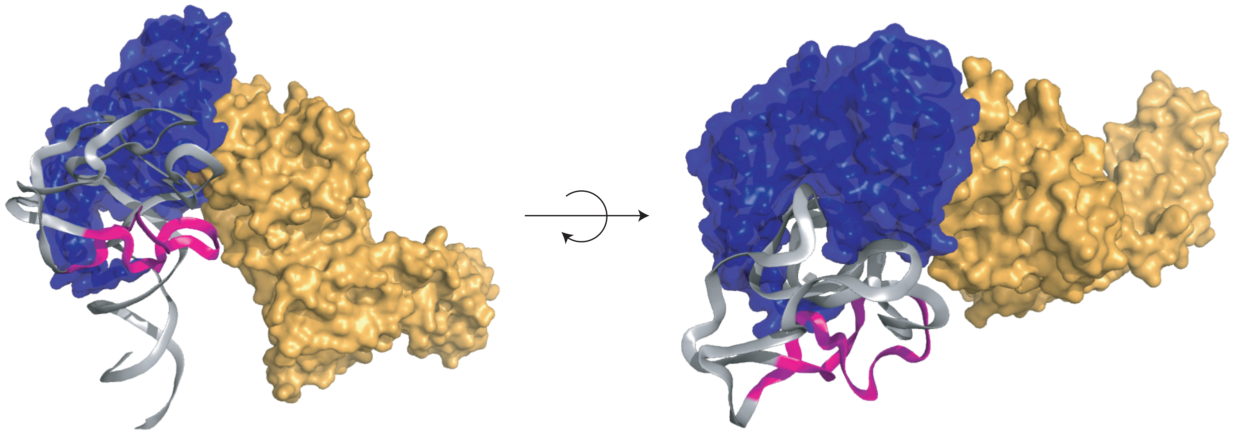

Supplementary Fig 6: Tertiary structure of L1 stalk (Silver), H78 (Purple), uL1 (Blue), and Der (Yellow). (PDB: 3J8G)

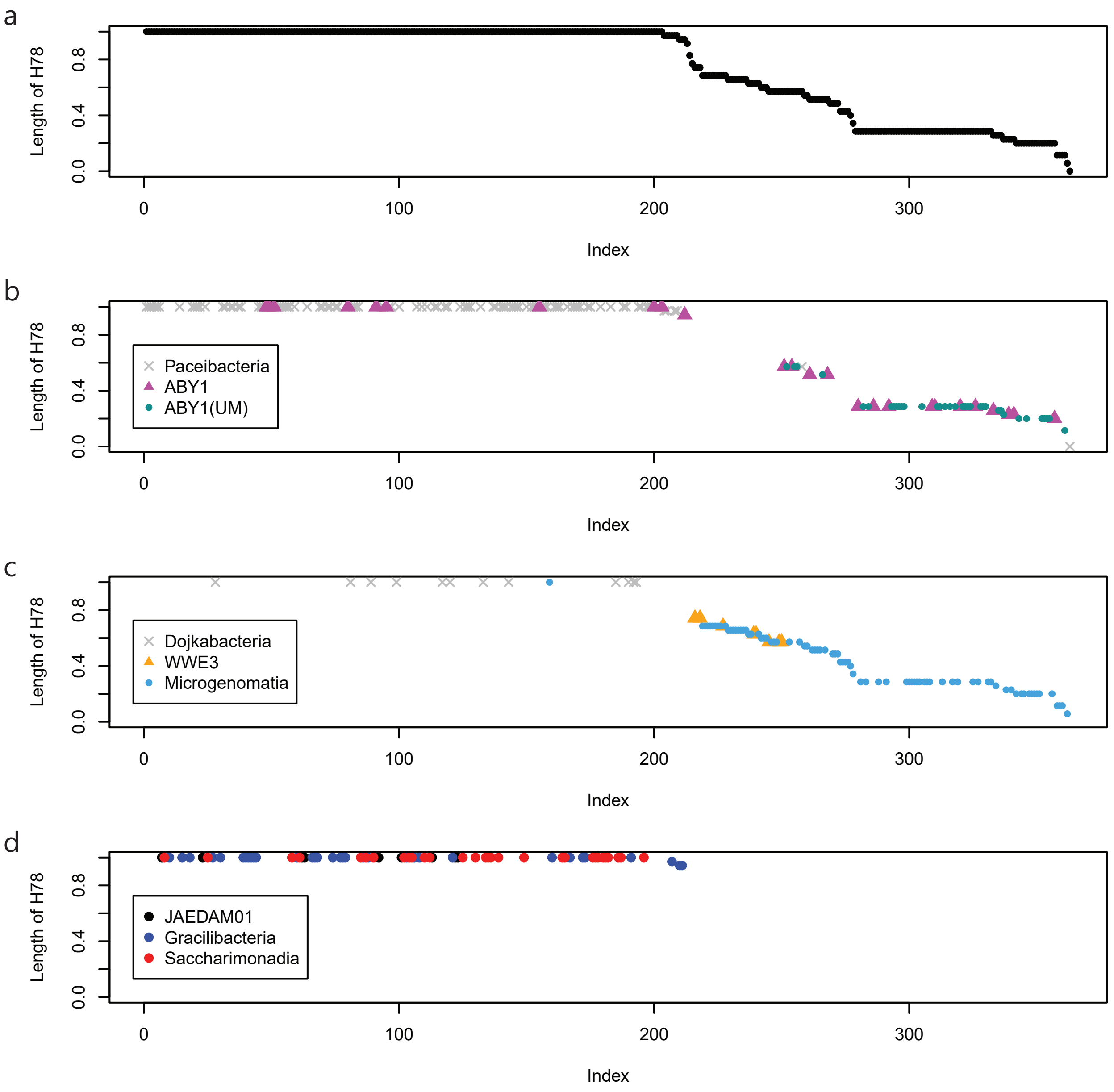

Supplementary Fig 7: a, The lengths of H78 length in CPR bacteria are arranged in order of length. **b,** in Paceibacteria, ABY1, and ABY1(UM) **c,** in Dojkabacteria, WWE3, and Microgenomatia **d,** in JAEDAM01, Gracilibacteria, and Saccharimonadia.

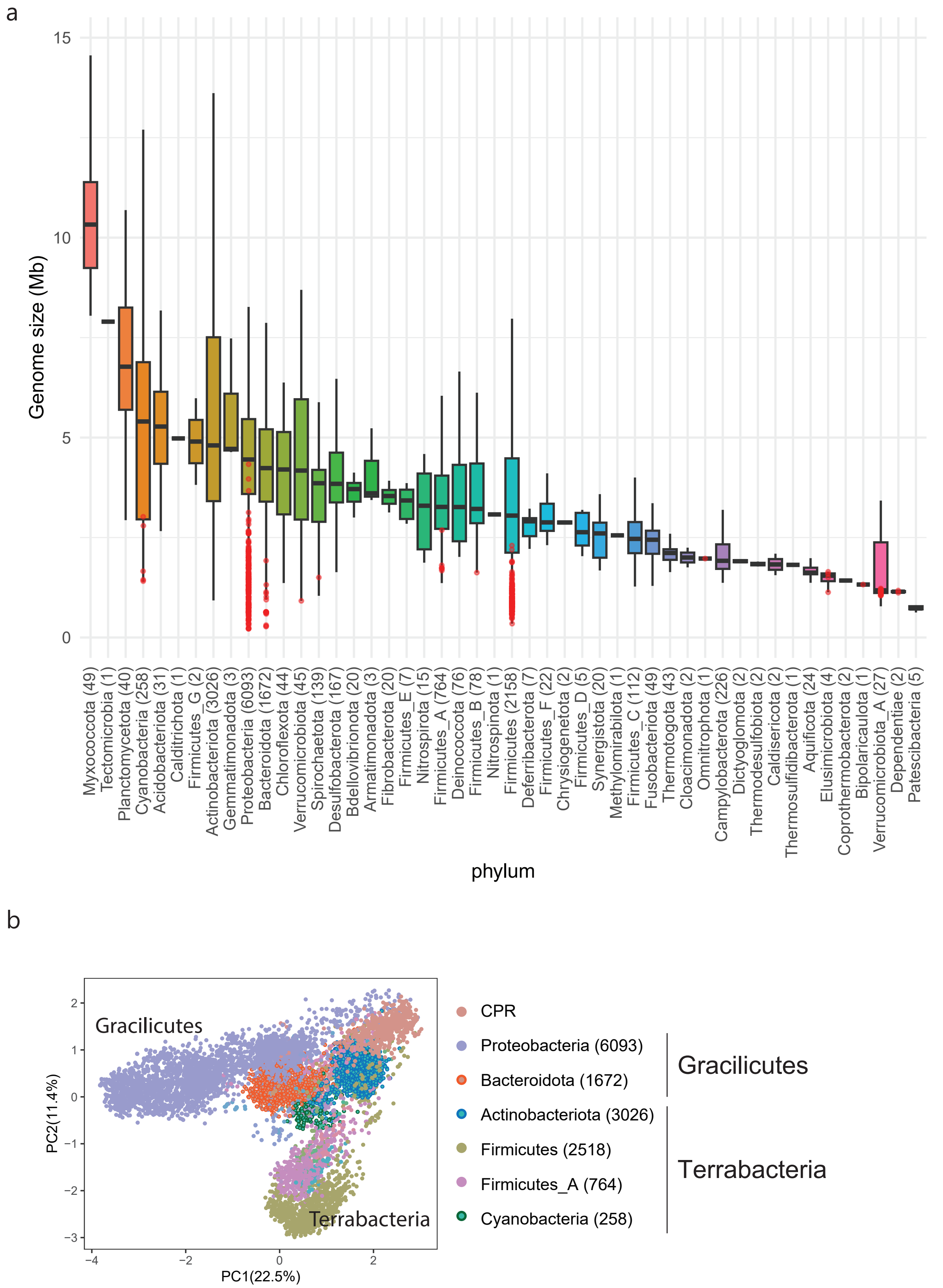

Supplementary Fig 8: Boxplot representation of the genome size across different phyla. a, Genomes in the AnnoTree with the completeness of 95 or more and registered for the RefSeq were selected. The red circle represents the genome size of symbiotic bacteria species. **b,** Colors of the plots in Figure 7C were modified, with the CPR and top six classes containing the highest number of species displayed in the legend.

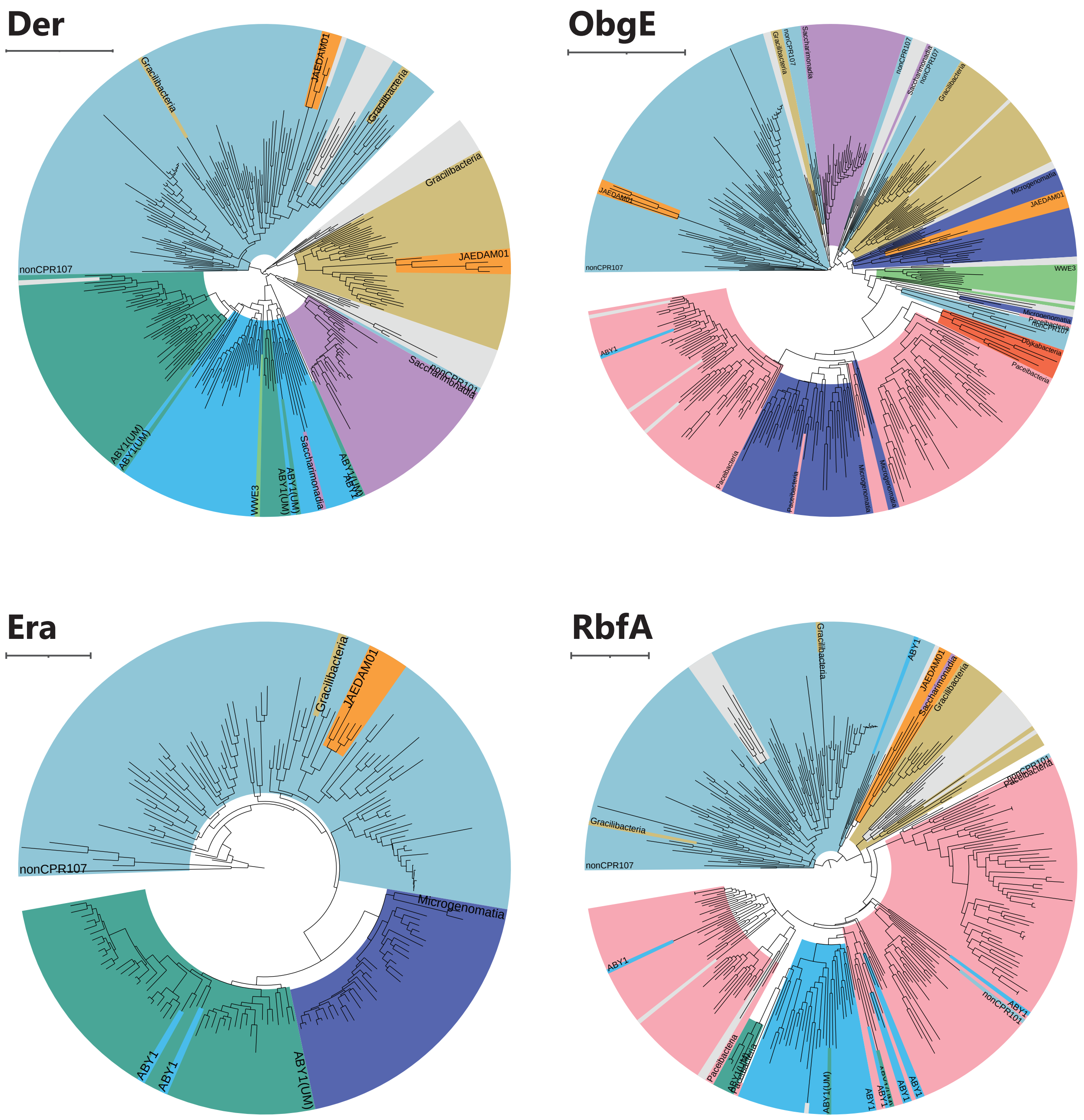

Supplementary Fig 9: Phylogenetic trees of four ribosome biogenesis factors (RBFs). The tree was constructed using IQ-TREE and rendered using iToL.
